# Single Crystalline Iron Silicide and Beta-Iron Disilicide Nanowires Formed through Chemical Vapor Deposition

**DOI:** 10.3390/ma11122384

**Published:** 2018-11-27

**Authors:** Wei-Jie Huang, Yu-Yang Chen, Hsiu-Ming Hsu, Kuo-Chang Lu

**Affiliations:** 1 Department of Materials Science and Engineering, National Cheng Kung University, Tainan 701, Taiwan; ecao1ah@hotmail.com (W.-J.H.); yuyang840228@gmail.com (Y.-Y.C.); hpolokoio@gmail.com (H.-M.H.); 2 Center for Micro/Nano Science and Technology, National Cheng Kung University, Tainan 701, Taiwan

**Keywords:** β-FeSi_2_, FeSi, chemical vapor deposition, nanowires, transmission electron microscopy

## Abstract

In this paper, we report the synthesis of iron silicide and β-iron disilicide nanowires with chemical vapor deposition; remarkably, the latter has drawn much attention but has seldom been achieved. We also propose the formation mechanisms for the two phases. To investigate the effects of the growth parameters on compositions and morphologies of the iron silicide nanowires, we changed and studied the reaction time, substrate temperature, position of samples, and pressure. The reaction concentration was found to be altered by all of the parameters; thus, we observed different nanowires in terms of morphologies and compositions with scanning electron microscopy. To confirm the growth direction and crystal structure of the nanowires, we conducted x-ray diffraction and high-resolution transmission electron microscopy studies. With the potential of being utilized as circuit elements in electronic devices for Schottky barriers, ohmic contacts, and interconnection among silicon-based transistors, the silicide work at nanoscale is beneficial for nanoelectronics. Understanding the effects of these growth parameters facilitates the control of nanowire growth with better quality.

## 1. Introduction

For the miniaturization of electronic devices, various materials are being studied for their promising applications. Graphene-ZnO nanoparticles were investigated for being applied in light-emitting diodes and photodetectors [1]. Fe^3+^-doped PbTiO_3_ nanocrystals were studied systematically for better understanding of this novel material [2]. Growing various iron oxides’ thin films with different precursors via the chemical vapor deposition (CVD) method was conducted [3]. Metal silicide technology has also attracted lots of interest for their distinct physical properties, as compared with materials in bulk and thin film. Transition-metal silicides have been studied extensively [4,5,6,7,8,9,10]. NiSi, TiSi_2_, CoSi_2_ and their excellent properties, such as low resistivity, high melting-point, and good stability, are generally used for metal-gate materials to reduce resistance in virtue of their low resistivity [11]. MnSi, β-FeSi_2_, and CrSi_2_ are great thermoelectric materials as a result of their good thermostability and narrow energy gap [12]. Metal silicide thin films can also be applied in electronic junctions and interconnecting devices. There are different metals that can be used for silicide applications based on their different properties, such as their silicidation temperatures, resistivities, dominant-diffusing species, and etching capabilities. 

One-dimensional nanostructures are promising for bottom-top microelectronics devices due to their novel properties, such as electrical properties, low defect density, and high compatibility [13], emerging as materials for academic study and technological applications [14]. Methods of nanowire synthesis have been studied for years. There are many approaches for synthesizing metal silicide nanowires (NWs). For example, NiSi NWs were synthesized by the chemical reaction between silicon NWs and nickel NWs. Nickel silicide NWs were also obtained by decomposition of silane on nickel substrates [15]. Chemical vapor deposition is another well-known method; although the reaction may seem simple, professional experiences are necessary for choosing appropriate precursors and controlling the reaction conditions; the morphology of the materials is very sensitive to temperature and the deposition position is a function of the flow rate, deposition temperature, and pressure. In recent years, growth of a variety of metal silicide NWs, ranging from transition metal silicides to rare-earth silicides, has been reported [16,17,18,19,20]. These silicide NWs were grown by first depositing an appropriate metal film on a Si substrate, followed by heat treatment. On the other hand, some growth methods have been reported for forming free-standing silicide NWs. Ouyang et al. used FeCl_3_ powders to grow ε-FeSi NWs on a Si wafer in an alumina tube furnace via Vapor-Phase Synthesis [21]. In addition, Schmitt et al. used a new method to produce free-standing, single-crystal FeSi NWs through organometallic precursor trans-Fe(SiCl_3_)_2_(CO)_4_ in a simple CVD process [22].

In this work, we demonstrate the synthesis of single-crystalline FeSi and β-FeSi_2_ nanowires by CVD. Iron disilicide has been studied recently since there are numerous specific phases in iron disilicide. α-FeSi_2,_ which is stable at temperatures ranging from 950 °C up to about 1250 °C, has a tetragonal structure with lattice parameters of a = b = 2.684 Å and c = 5.124 Å, possessing a metallic property. β-FeSi_2_ has an orthorhombic structure with lattice parameters of a = 9.863 Å, b = 7.791 Å, and c = 7.833 Å, being a semiconductor stably below 950 °C [23]. In addition to these two stable phases, there are two other metastable metallic FeSi_2_ phases. γ-FeSi_2_, is metallic and magnetic, having a FCC structure with lattice parameters of a = 5.389 Å. Although s-FeSi_2_ is metallic structured, it has a BCC structure with lattice parameters of a = 2.7 Å [24,25,26,27]. FeSi is a narrow-band gap semiconductor with a cubic structure, where a = b = c = 4.485 Å, and has been classified as a Kondo insulator. 

The unusual features of β-FeSi_2_ is of particular interest. β-FeSi_2_ nanowires and their applications on communication were reported [28]; however, there have been difficulties in forming β-FeSi_2_ nanowires through chemical vapor deposition. The room-temperature equilibrium phase, β-FeSi_2_, has a direct band gap, making it a promising choice in infrared detectors and light emitters for silicon-based optoelectronics. Recently, the use of magnetic semiconducting silicide, Fe_1−x_Co_x_Si, as injectors for spintronics applications has been reported [29]. In this study, we report efficient synthesis and structural characterization of the single crystalline β-FeSi_2_ nanowires via chemical vapor deposition. 

## 2. Materials and Methods

**Figure 1:**
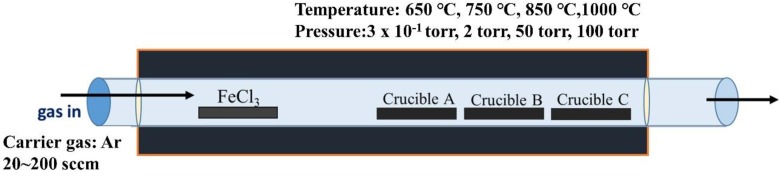
Schematic illustration of the CVD system, with FeCl_3_ as precursors.

Figure 1 is the schematic illustration of the experimental procedures. In this work, the synthesis of β-FeSi_2_ and FeSi nanowires was conducted in a conventional three-zone furnace through chemical vapor deposition. The furnace has a structure of double-layer quartz tubes; the diameter of the inner tube is 50 mm, while that of the outer tube is 75 mm, and both tubes are 120 cm long. Firstly, single-crystalline Si (100) substrates were cut into 2 cm × 2 cm and were cleaned in acetone and isopropyl alcohol with ultrasonication. To remove the native oxide layer, substrates were dipped in dilute HF for 5 min; then, they were put into crucibles A, B, and C. Crucible A was located in the middle of the second heating zone, and crucible C was in the middle of the third heating zone. Crucible B was between crucible A and C. Anhydrous iron chloride powders, as precursors in our work, were placed in an alumina boat, upstream of the first heating zone. The distance away from the precursor for crucibles A, B, and C was 20, 30, and 40 cm, respectively. To understand the factors that influence the growth of iron silicide nanowires, processing parameters, including the temperature, pressure, and gas flow-rate were varied and studied. We utilized X-ray diffraction (XRD, D8 discover with GADDS, Bruker AXS Gmbh, Karlsruhe, Germany) and transmission electron microscope (TEM, JEM-1400, JOEL, Akishima, Japan) for structural characterization and composition analysis. Field emission scanning electron microscope (FESEM, SU8000, Hitachi, Tokyo, Japan) was utilized for the morphology. 

## 3. Results and Discussion

### 3.1. Morphology 

#### 3.1.1. Temperature

**Figure 2:**
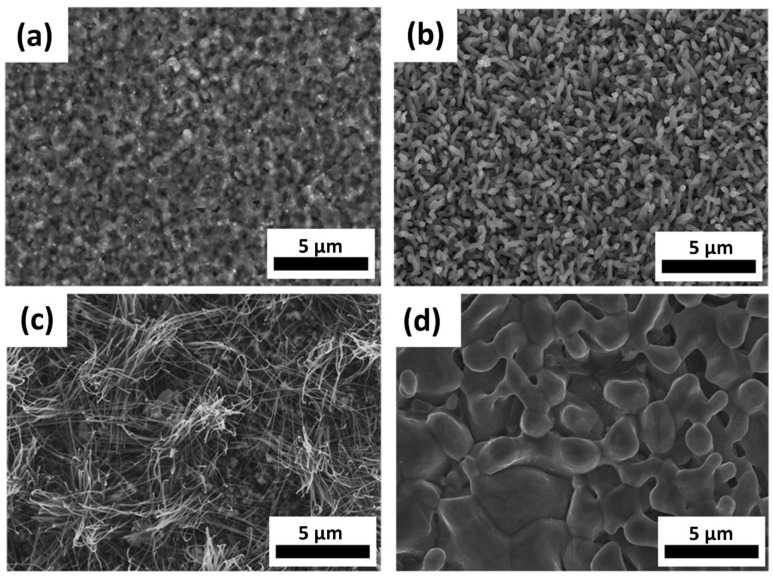
SEM images of samples grown at (**a**) 650 °C, (**b**) 750 °C, (**c**) 850 °C, and (**d**) 1000 °C at 3 × 10^−1^ torr for 2 h.

All samples were collected from crucible B. Figure 2a–d shows the SEM images of samples grown at different temperatures (650 °C, 750 °C, 850 °C, and 1000 °C, respectively) for 2 h, where the ambient pressure was 3 × 10^−1^ torr, and the flow rate was fixed at 20 sccm. Figure 2a shows the case at 650 °C, where no NWs but thin films were grown on the substrate. Figure 2b shows the case at 750 °C, where the NWs were short in length with typical diameters ranging from 70 to 100 nm. When we raised the temperature to 850 °C as shown in Figure 2c, NWs with the highest aspect ratio and of about 20 to 40 nm in diameter were obtained. Figure 2d shows the case at 1000 °C, where thin films with coarse grains were found on the substrates. At high temperatures, it was easy for the precursor to transform into the vapor phase and be transported to the crucibles; thus, the reaction concentration was increased. With high reaction concentration, the growth rate was fast, changing the morphology of the samples from nanowires to thin film. On the other hand, the reaction rate was slow at 650 °C, and the duration time of 2 h was too short for NWs growth; as a result, no nanowires appeared at 650 °C. Therefore, we found that the best growth temperatures here were 700–850 °C. 

#### 3.1.2. Pressure and Flow Rate

**Figure 3:**
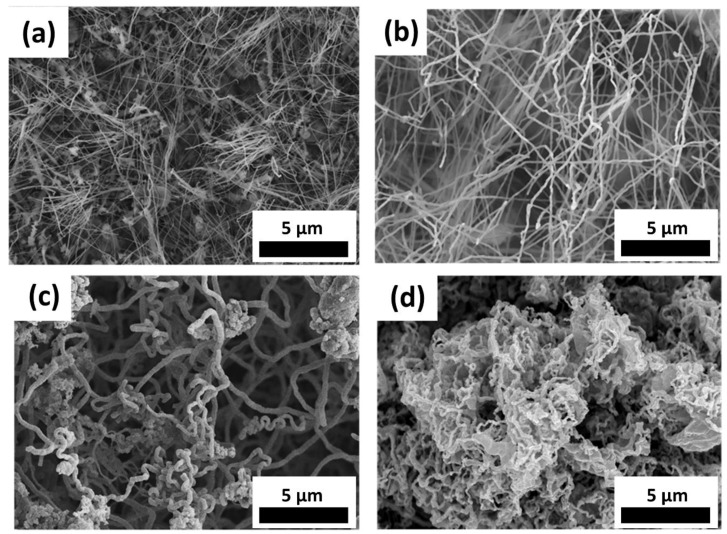
SEM images of samples grown at: (**a**) 3 × 10^−1^ torr, (**b**) 2 torr, (**c**) 50 torr, and (**d**) 100 torr.

The second parameter we investigated was the pressure. Figure 3a–d shows the SEM images for 3 × 10^−1^, 2, 50, and 100 torr, respectively. While we conducted the experiment, the temperature was kept at 850 °C for 2 h, and the gas flow-rate was 20 sccm. The diameter of NWs grown at 3 × 10^−1^ torr was 20 to 40 nm, while that of nanowires grown at 100 torr was 100 to 150 nm with the decrease of the density. The diameter of the NWs increased as the pressure was increased. This was due to the fact that at high ambient pressure, the vapor pressure of the precursor was low, which caused the lower reaction concentration. In our experiment, the pressure was controlled by the partial pressure of Ar, and the effect of ambient pressure could be explained by Rault’s law:

(1) $$P=P_{\mathrm{Ar}}^{*}\times X_{\mathrm{Ar}}+P_{{\mathrm{FeCl}}_{3}}^{*}\times X_{{\mathrm{FeCl}}_{3}}$$

(2) $$\Delta P_{{\mathrm{FeCl}}_{3}}=P_{{\mathrm{FeCl}}_{3}}^{*}\times X_{{\mathrm{FeCl}}_{3}}$$

where P is the ambient pressure in the furnace, $P_{\mathrm{Ar}}^{*}$ is the saturated vapor pressure of Ar, $P_{{\mathrm{FeCl}}_{3}}^{*}$ is the saturated vapor pressure of FeCl_3_, $X_{\mathrm{Ar}}$ is the mole fraction of Ar, $X_{{\mathrm{FeCl}}_{3}}$ is the mole fraction of FeCl_3_, and $\Delta P_{{\mathrm{FeCl}}_{3}}$ is the change of the partial pressure of FeCl_3_. In our study, the increase of the ambient pressure was achieved by the large quantity of Ar. Therefore, the mole fraction of FeCl_3_ and its partial pressure decreased with the increase of Ar. Through ideal gas function, the gas concentration is directly proportional to the pressure. The decrease of FeCl_3_ vapor pressure suggested that the concentration of FeCl_3_ became lower. The free energy of nucleation is a function of the reaction concentration.

(3) $$\Delta G=-\frac{\mathrm{kT}}{\Omega }\ln\left(\frac{C}{C_{0}}\right)$$

where ΔG is the free energy for nucleation, T is the temperature, Ω is the atomic volume, C is the reaction concentration, and C_0_ is the equilibrium reaction concentration. The decrease of the reaction concentration reduced the free energy for nucleation in FeCl_3,_ which increased the critical size for nucleation.

(4) $$r^{*}=\frac{-2\gamma }{\Delta G^{*}}$$

$r^{*}$ is the critical radius of nuclei, γ is the surface energy, and $\Delta G^{*}$ is the critical energy for nucleation. As the ambient pressure increased, the reaction concentration was lower and the size of the nuclei became larger, which explains why NWs grown at high ambient pressure possess wider diameters. 

**Figure 4:**
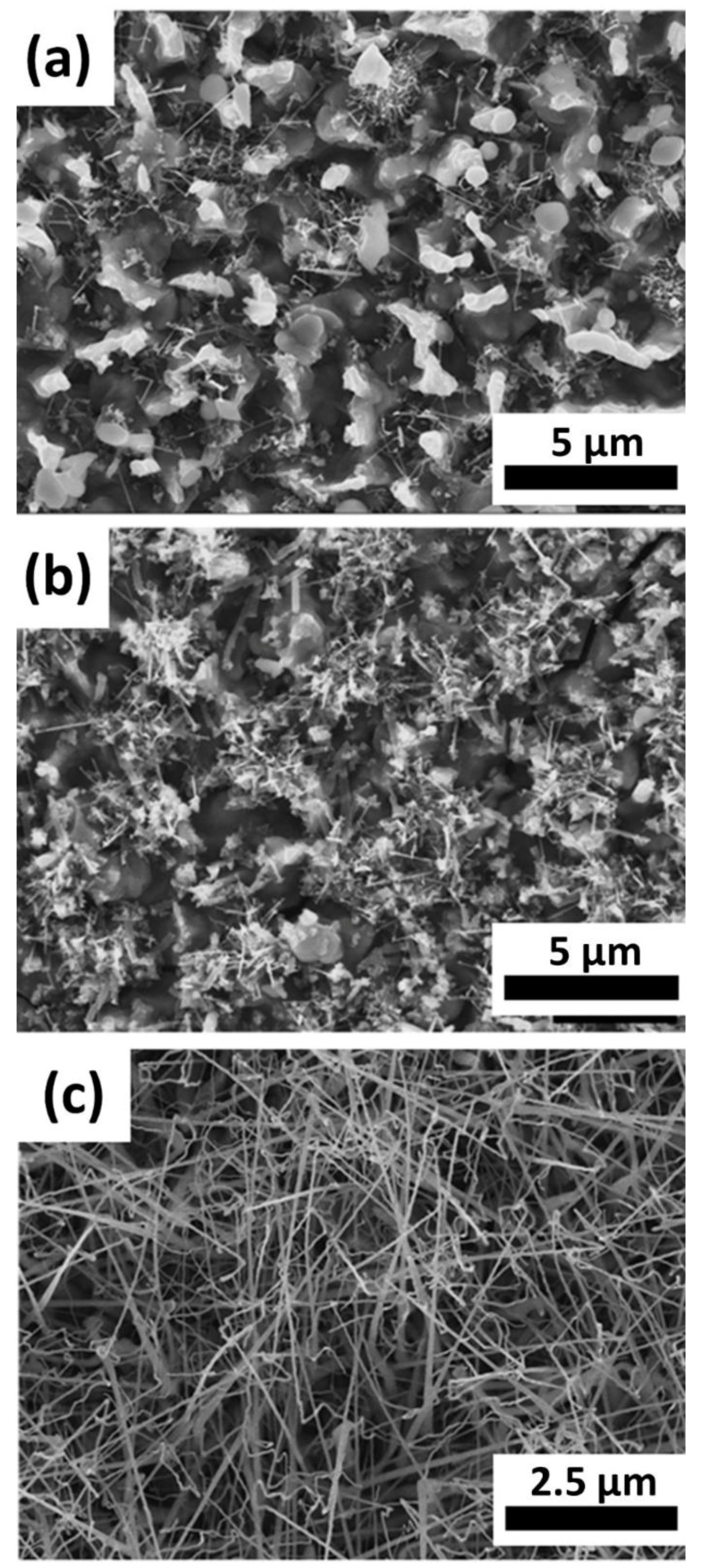
SEM images of nanowires grown at gas flow rates (**a**) 100 sccm and (**b**) 200 sccm at 850 °C. (**c**) SEM image of nanowires grown at 1000 °C for 5 min.

Gas flow rate and deposition position are important factors in CVD [30], which would directly affect the distribution of reaction concentration. Figure 4a, b show the morphology of samples grown at 850 °C for 2 h at the flow rate of 100 and 200 sccm, respectively. At high flow rate, most vapor molecules of the precursor were taken away from the quartz tube, and the reaction concentration in the quartz tube was low. Thus, less precursor molecules deposited onto the silicon substrates, contributing to less NW growth. Figure 4c shows NWs grown at 1000 °C for 5 min. If we compare with Figure 2d, reducing the reaction time at 1000 °C would prevent the overreaction of the growth of NWs because the reaction rate at 1000 °C was very fast.

### 3.2. Structure Analysis

**Figure 5:**
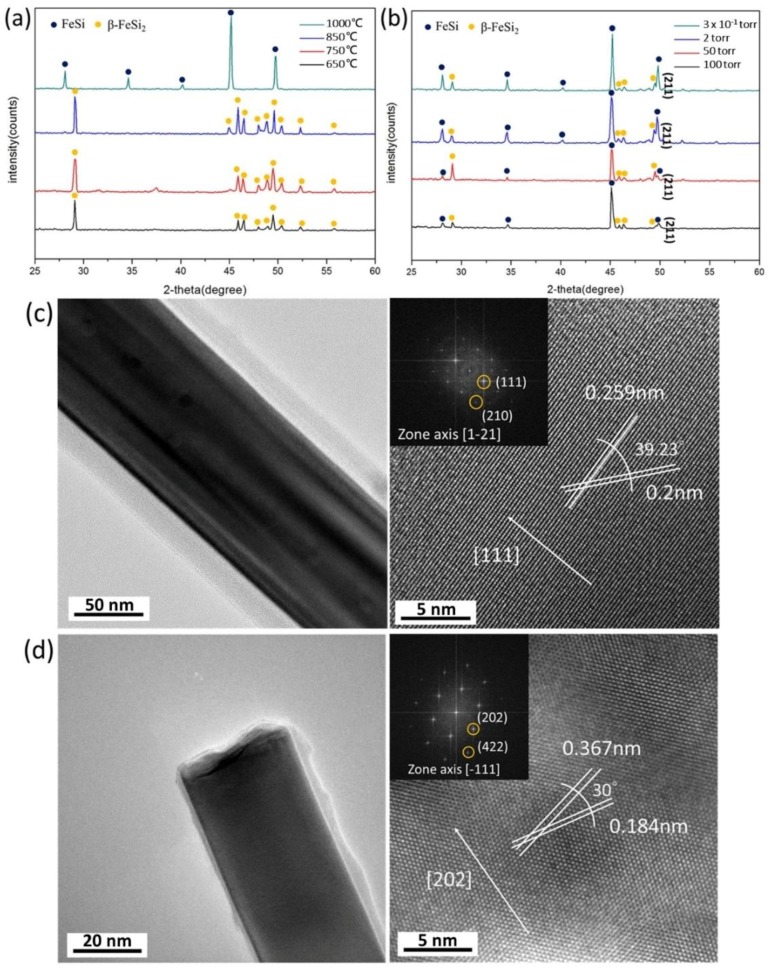
(**a**) XRD spectrums of β-FeSi_2_ and FeSi at different growth temperatures. (**b**) XRD spectrums of β-FeSi_2_ and FeSi at different ambient pressures. (**c**,**d**) Low-magnification TEM and HR-TEM images of FeSi and β-FeSi_2_ nanowires (NWs). (**c**) FeSi nanowires grown at 1000 °C for 5 min. The inset shows the corresponding diffraction patterns of FeSi. (**d**) β-FeSi_2_ nanowires grown at 850 °C for 2 h. The inset shows the corresponding diffraction patterns of β-FeSi_2_.

Figure 5a–d are XRD spectra and TEM images of the samples grown at different temperatures and pressures. Figure 5a, the XRD analysis, indicates that FeSi was formed at 1000 °C, while β-FeSi_2_ was formed at 650 °C, 750 °C, and 850 °C. These results are consistent with previous studies [21,31]. Figure 5b is the spectrum of NWs grown at different ambient pressures. As the pressure increased, the intensity of the characteristic peak (211) was reduced, which suggested the content of FeSi was lower at a higher pressure. Figure 5c,d are a low-magnification TEM image, HR-TEM image, and diffraction pattern of nanowires grown at 1000 °C for 5 min and 850 °C for 2 h, respectively. Through the TEM analysis, the growth direction, which was [111] for FeSi and [202] for β-FeSi_2,_ and the lattice structures, were confirmed. At higher temperatures, NWs were single crystalline, while at lower temperatures, the crystallinity of the NWs was worse. Additionally, we found that the impact of the temperature was larger than that of the pressure. 

### 3.3. Growth Mechanism 

**Figure 6:**
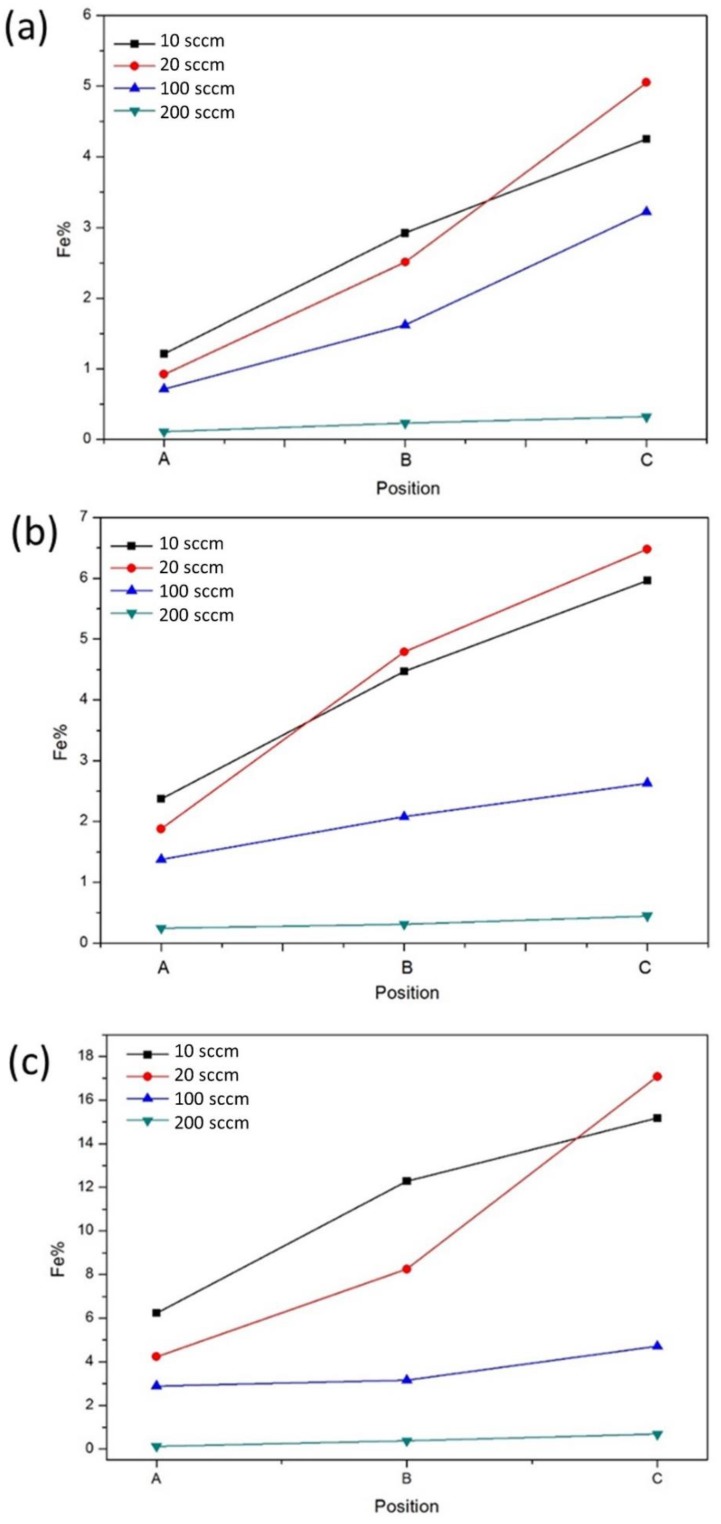
The EDX analysis of Fe on the substrate for different temperatures: (**a**) 650 °C, (**b**) 850 °C, and (**c**) 1000 °C.

**Figure 7:**
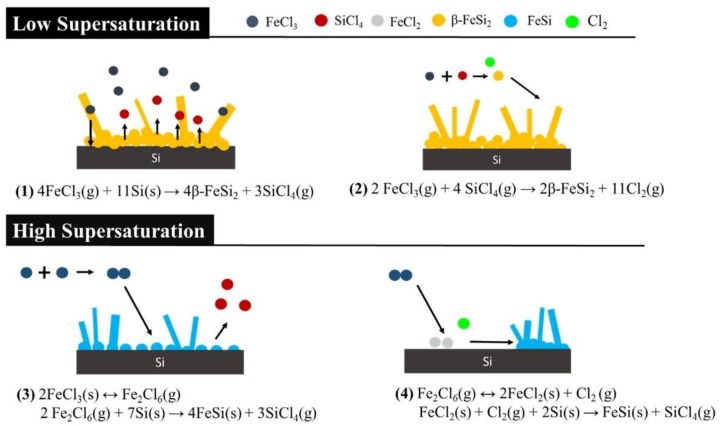
The growth mechanism of iron silicide nanowires.

In order to observe the concentration distribution in our experiment and connect the concentration to processing parameters and the phase that would form in the experiment, a method was utilized here. First, Si (100) substrates were replaced by Al_2_O_3_ substrates, and the substrates were put into crucibles A, B, and C. Then, FeCl_3_ was deposited on Al_2_O_3_ substrates with different processing parameters. These Al_2_O_3_ substrates in the different crucibles were taken for element analysis by energy-dispersive X-ray spectroscopy (EDX, Bruker, Billerica, MA, USA), and the results are shown in Figure 6. The reaction concentration at 1000 °C was higher than that at 850 and 650 °C; we found that the reaction concentration became lower when the flow rate increased. The results indicate that FeSi NWs were grown at high temperature, while no NWs appeared at a high flow rate. As we considered different deposition positions, at high flow rates (100 and 200 sccm), the concentration distribution was uniform; however, at low flow rates (10 and 20 sccm), the reaction concentration was higher in the crucible which was further away from the precursor. This may be attributed to the fact that in the experiment, the pump pulled out the gas in the quartz tube, making most of the FeCl_3_ molecules brought to the downstream. Thus, the probability for the precursor to deposit on the substrate in crucible C increased. The reactions of FeCl_3_ with silicon substrates to form β-FeSi_2_ and FeSi nanowires may follow the reaction pathways below, and the growth mechanisms are shown in Figure 7.

β-FeSi_2_:

4FeCl_3_(g) + 11Si(s) → 4β-FeSi_2_ + 3SiCl_4_(g) (5)

2FeCl_3_(g) + 4SiCl_4_(g) → 2β-FeSi_2_ + 11Cl_2_(g) (6)

FeSi:

2FeCl_3_(s) ↔ Fe_2_Cl_6_(g) (7)

2Fe_2_Cl_6_(g) + 7Si(s) → 4FeSi(s) + 3SiCl_4_(g) (8)

Fe_2_Cl_6_(g) ↔ 2FeCl_2_(s) + Cl_2_ (g) (9)

FeCl_2_(s) + Cl_2_(g) + 2Si(s) → FeSi(s) + SiCl_4_(g) (10)

Through the reaction concentration analysis above, we found that a higher temperature at a lower flow rate would cause a higher concentration. The reaction concentration was the key to deciding which composition was formed. FeSi nanowires were fabricated in a high reaction concentration environment, while β-FeSi_2_ nanowires were fabricated in a low one. Lots of experimental evidence indicates that the mechanism of the nanowires’ fabrication is a vapor-solid mechanism [32]. In our case, there were no catalyst particles or metal liquid droplets found on the tip of the nanowires, suggesting that the growth mechanism in our experiment was not a vapor-liquid-solid mechanism. There were several steps in the growth process of nanowires. First, the FeCl_3_ particles were deposited on the Si substrate, and formed silicide particles. Second, these particles agglomerated and formed thin films. Then, nanowires were grown from the heterogeneous nucleation sites on the surface of the thin films. 

## 4. Conclusions

In this study, we synthesized two phases of iron silicide NWs via the CVD method, which were FeSi and β-FeSi_2._ The influence of processing parameters, such as temperature, ambient pressure, and gas flow rate were systematically investigated, and the growth mechanism was also proposed. The phase of NWs grown at 1000 °C was FeSi, while that grown at 850 °C was β-FeSi_2_. The diameter of NWs became wider with the increase of ambient pressure. Gas flow rate affected the number and density of the NWs grown on the substrates; with a higher flow rate, the number of the NWs was reduced. The reason why these parameters influenced the results of our experiment was that all of them varied the reaction concentration in the furnace. A high temperature, low ambient pressure, and low gas flow rate would create an environment of high reaction concentration; the reaction time should be shortened to grow NWs on the substrate.
